# Incidence Trends of Breast Cancer Molecular Subtypes by Age and Race/Ethnicity in the US From 2010 to 2016

**DOI:** 10.1001/jamanetworkopen.2020.13226

**Published:** 2020-08-17

**Authors:** Teofilia Acheampong, Rebecca D. Kehm, Mary Beth Terry, Erica Lee Argov, Parisa Tehranifar

**Affiliations:** 1 Mailman School of Public Health, Department of Epidemiology, Columbia University, New York, New York; 2 Herbert Irving Comprehensive Cancer Center, Columbia University Medical Center, New York, New York

## Abstract

**Importance:**

Breast cancer incidence trends by age and race/ethnicity have been documented; it is less clear whether incidence trends of breast cancer molecular subtypes, which differ in risk factors and prognosis, also vary by age and race/ethnicity.

**Objective:**

To estimate annual percentage changes and trends in breast cancer molecular subtype–specific incidence rates by age at diagnosis and race/ethnicity in the US.

**Design, Setting, and Participants:**

This population-based cross-sectional study included data from 18 cancer registries in the Surveillance, Epidemiology and End Results database, capturing 27.8% of the US population. Hispanic and non-Hispanic White, Black, and Asian/Pacific Islander women aged 25 to 84 years who were diagnosed with invasive breast cancer from 2010 to 2016 were included. Data were analyzed from September 2019 to February 2020.

**Exposures:**

Age and racial/ethnic groups.

**Main Outcomes and Measures:**

Annual percentage change (APC) and 95% CIs for age-standardized breast cancer incidence rates stratified by 15-year age groups at diagnosis and race/ethnicity.

**Results:**

Of 320 124 women diagnosed with breast cancer from 2010 to 2016, 232 558 (72.6%) had luminal A, 35 869 (11.2%) had luminal B, 15 472 (4.8%) had ERBB2-enriched, and 36 225 (11.3%) had triple-negative breast cancer subtypes. Luminal A breast cancer incidence rates increased in non-Hispanic White (APC from 2010-2014, 2.3%; 95% CI, 0.3% to 4.2%) and non-Hispanic Asian/Pacific Islander (APC from 2010-2016, 2.5%; 95% CI, 0.6% to 4.5%) women aged 40 to 54 years, and in non-Hispanic Black women aged 55 to 69 years women (APC from 2010-2012, 4.9%; 95% CI, 4.0% to 5.7%). Luminal B breast cancer incidence rates increased in all age groups for non-Hispanic White women (age 25-39 years: APC, 4.3%; 95% CI, 1.5% to 7.%2; age 40-54 years: APC, 3.5%; 95% CI, 1.4% to 5.6%; age 55-69 years: APC, 3.3%; 95% CI, 1.6% to 5.0%; age 70-84 years: APC, 3.9%; 95% CI, 1.9% to 6.0%) and Hispanic women (age 25-39 years: APC, 8.4%; 95% CI, 5.8% to 11.2%; age 40-54 years: APC, 6.1%; 95% CI, 4.2% to 8.0%; age 55-69 years: APC, 5.1%; 95% CI, 1.5% to 8.8%; age 70-84 years: APC, 7.1%; 95% CI, 4.6% to 9.6%) and in non-Hispanic Asian/Pacific Islander women aged 55 to 69 years (APC, 6.1%; 95% CI, 3.2% to 9.0%). ERBB2-enriched breast cancer incidence rates increased in non-Hispanic White women aged 25 to 39 years (APC, 4.7%; 95% CI, 1.5% to 8.0%). Triple-negative breast cancer incidence rates decreased in non-Hispanic White women aged 40 to 54 years (APC, –2.3%; 95% CI, –3.8% to –0.7%) and 55 to 69 years (APC, –3.6%; 95% CI, –5.1% to –2.1%) and in non-Hispanic Black women aged 55 to 69 years (APC, –1.4%; 95% CI, –2.2% to –0.7%).

**Conclusions and Relevance:**

The findings of this cross-sectional study suggest that between 2010 and 2016, luminal A and luminal B breast cancer incidence rates increased for many racial/ethnic and age groups, with the largest increases observed for luminal B breast cancer. ERBB2-enriched breast cancer incidence rates increased for young non-Hispanic White women, while triple-negative breast cancer incidence rates decreased for midlife non-Hispanic White and non-Hispanic Black women. These trends may suggest changes in breast cancer risk factor profiles across age and racial/ethnic groups.

## Key Points

**Question:**

Are incidence rates of US breast cancer molecular subtypes changing across age and racial/ethnic groups?

**Findings:**

In this cross-sectional study including 320 124 women diagnosed with breast cancer from 2010 to 2016, incidence rates of luminal A breast cancer increased in non-Hispanic White and Asian/Pacific Islander women aged 40 to 69 years, and in non-Hispanic black women aged 55 to 69 years, for luminal B breast cancer in non-Hispanic White and Hispanic women of all ages, and for ERBB2-enriched breast cancer in non-Hispanic White women aged 25 to 39 years. Incidence rates for triple-negative breast cancer decreased in non-Hispanic Black and non-Hispanic White women aged 40 to 69 years.

**Meaning:**

These findings of differing breast cancer molecular subtype–specific trends may suggest changes in the prevalence of breast cancer risk factors by race/ethnicity and age.

## Teaser

This cross-sectional study examines the incidence of different molecular subtypes of breast cancers among different racial/ethnic and age groups.

## Introduction

Population-based studies suggest a mostly stable trend in overall breast cancer incidence rates,^1,2^ although there are documented differences in trends by age at diagnosis and racial/ethnic groups.^1,3,4,5^ Moreover, studies have reported racial/ethnic differences in the incidence rates of breast cancer subtypes, defined according to hormone receptor status or ERBB2 (formerly HER2) receptor status, but these studies have mostly considered few broad molecular subtype, race/ethnicity, or age categories.^6,7,8^ A 2019 report^2^ of temporal changes in breast cancer incidence rates from 2004 through 2016, which used only breast cancer subtypes by hormone receptor status, found that incidence rates increased for hormone receptor–positive breast cancers (corresponding to both luminal A and B molecular breast cancer subtypes) for all races/ethnicities, although rates began to stabilized after 2011 for non-Hispanic Black and American Indian and Alaska Native women. In contrast, hormone receptor–negative tumors (corresponding to both ERBB2-enriched and triple-negative molecular breast cancer subtypes) decreased for women of all racial/ethnic groups by 1.5% to 2.6% per year. Furthermore, differences in trends for women by 2 broad age groups (<50 years vs ≥50 years), were similar, although not statistically significant for the hormone receptor–negative decline in young American Indian and Alaska Native or older Hispanic women.^2^ A previous cross-sectional study also examined breast cancer incidence rates by racial/ethnic and age group differences in 4 breast cancer molecular subtypes based on joint assessment of both hormone receptor status and ERBB2 status using 2010 cancer registry data.^6^ However, studies assessing trends in the incidence rates of breast cancer subtypes are limited, as ERBB2 status was only required to be collected by cancer registries starting in 2010.

The heterogeneity of breast cancer at the molecular level is etiologically and clinically meaningful, as it maps to distinct risk factors as well as to differences in treatment effectiveness and prognosis.^2,9,10,11^ Furthermore, the disproportionate distribution of molecular subtypes by race/ethnicity may partially account for racial/ethnic disparities in breast cancer outcomes, particularly among younger women.^12,13^ Therefore, breast cancer subtype-specific trends may reveal patterns with important implications for breast cancer etiology and reducing breast cancer health disparities.

We used data from US population-based Surveillance Epidemiology and End Results (SEER) cancer registries to examine trends in the incidence of breast cancer by age and racial/ethnic groups from 2010 to 2016 for 4 molecular subtypes, defined by joint expression of hormone receptor and ERBB2 status. This time period includes the most complete data on breast cancer molecular subtypes, including ERBB2 status, to our knowledge.

## Methods

### Study Population

We obtained data from the SEER 18 cancer registry database (November 2018 submission, 2000-2016),^14^ representing 12 states and capturing 27.8% of the US population. We filed a data-use agreement with the SEER National Cancer Institute for access to the deidentified database; therefore, the Columbia University institutional review board deemed this study exempt from approval and informed consent, per institutional policy. This study followed the Strengthening the Reporting of Observational Studies in Epidemiology (STROBE) reporting guideline.

We identified women aged 25 to 84 years who had been diagnosed with a primary invasive breast cancer (*International Classification of Diseases for Oncology, Third Edition* [*ICD-O-3*]^15^: C50.0 to C50.9) from 2010 to 2016. We excluded women with tumors that were of unknown molecular subtype or not microscopically confirmed. We focused on the largest racial/ethnic subgroups, excluding the American Indian and Native Alaskan group owing to having fewer than 5 cases by subtype and age group per year for Joinpoint analyses.

We categorized age group at diagnosis based on 15-year intervals (ie, ages 25-39, 40-54, 55-69, and 70-84 years) and categorized race/ethnicity into 4 mutually exclusive groups: non-Hispanic White, non-Hispanic Black, non-Hispanic Asian/Pacific Islander, and Hispanic ethnicity (97.1% of whom were Hispanic White). We used SEER’s breast subtype variable,^16^ which groups tumors into 4 breast cancer subtype categories based on joint expressions of hormone receptors capturing estrogen and progesterone receptors and with or without the ERBB2 marker: luminal A, with hormone receptor expression and without ERBB2 expression; luminal B, with hormone receptor and ERBB2 expressions; ERBB2-enriched, without hormone receptor expression and with ERBB2 expression; and triple negative, without hormone receptor or ERBB2 expressions.^6^

### Statistical Analysis

We calculated annual age-standardized breast cancer incidence rates per 100 000 women and SEs using SEER*Stat software version 8.3.6.^17^ We also calculated changes in trends using the National Cancer Institute’s Joinpoint software version 4.7.0.0.^18^ We fit weighted least-squares regression models, with a log-linear function, to estimate change in age-standardized annual incidence rates for each population subgroup, defined by age and racial/ethnic groups. We defined the independent variable as the year of diagnosis, and a maximum of 1 Joinpoint was automatically set based on the number of years. The selection of final models was based on a series of permutation tests and an overall α level of 0.05. We estimated the annual percentage change (APC) from the slope of the final model and corresponding 95% CIs. Statistically significant APCs were based on 2-sided tests using *t* distribution. Data were analyzed from September 2019 to February 2020.

## Results

Among 348 586 women with breast cancer in the SEER database, we excluded 26 520 women with tumors of unknown molecular subtype and 82 women with tumors that were not microscopically confirmed. We excluded 1860 American Indian and Native Alaskan women owing to having fewer than 5 cases by subtype and age group per year. The final sample comprised 320 124 women (91.8% of original sample) diagnosed with incident breast cancer during 2010 to 2016. Most women were non-Hispanic White (216 092 women [67.5%]) and aged 55 to 69 years at diagnosis (132 986 women [41.5%]) (Table 1). A total of 232 558 tumors (72.6%) were Luminal A, 36 225 tumors (11.3%) were triple negative, 35 869 tumors (11.2%) were luminal B, and 15 472 tumors (4.8%) were ERBB2-enriched. Missing data on molecular subtype were more common in Hispanic women overall and women aged 70 to 84 years in each race/ethnic group (eTable in the Supplement).^5^

**Table 1.** Number and Incidence Rates of Breast Cancer Per 100 000 Women Stratified by Race/Ethnicity, Age-Group and Molecular Subtype From 2010 to 2016

Subtype	Non-Hispanic White (n = 216 092)		Non-Hispanic Black (n = 36 267)		Non-Hispanic Asian/Pacific Islander (n = 29 309)		Hispanic (n = 38 456)	
	No. (%)	Rate^a^ (95% CI)	No. (%)	Rate^a^ (95% CI)	No. (%)	Rate^a^ (95% CI)	No. (%)	Rate^a^ (95% CI)
Age 25-39 y								
Luminal A^b^	4950 (54.5)	17.9 (17.4-18.4)	1300 (48.3)	17.8 (17.0-18.9)	1307 (59.9)	17.6 (16.7-18.6)	1897 (51.9)	12.6 (12.1-13.2)
Luminal B^c^	1845 (20.3)	6.6 (6.3-6.9)	491 (18.2)	6.7 (6.1-7.3)	405 (18.6)	5.4 (4.9-6.0)	648 (17.7)	4.3 (4.0-4.6)
ERBB2 enriched^d^	676 (7.4)	2.4 (2.2-2.6)	192 (7.1)	2.6 (2.3-3.0)	169 (7.7)	2.3 (1.9-2.6)	299 (8.2)	2.0 (1.8-2.2)
Triple negative^e^	1611 (17.7)	5.7 (5.4-6.0)	711 (26.4)	9.7 (9.0-10.5)	301 (13.8)	4.0 (3.6-4.5)	814 (22.3)	5.4 (5.0-5.7)
Age 40-54 y								
Luminal A^b^	41 930 (71.6)	113.3 (112.2-114.5)	6783 (56.5)	84.4 (82.4-86.5)	7564 (69.5)	102.7 (100.4-105)	9855 (65.6)	75.7 (74.2-77.2)
Luminal B^c^	7430 (12.7)	20.4 (20.0-20.9)	1597 (13.3)	19.9 (19.0-20.9)	1611 (14.8)	21.8 (20.8-22.9)	2175 (14.5)	16.7 (16.0-17.4)
ERBB2 enriched^d^	2826 (4.8)	7.6 (7.3-7.9)	842 (7.0)	10.4 (9.7-11.2)	809 (7.4)	10.9 (10.1-11.7)	965 (6.4)	7.4 (7.0-7.9)
Triple negative^e^	6351 (10.8)	17.5 (17.1-17.9)	2788 (23.2)	34.6 (33.3-35.9)	902 (8.3)	12.3 (11.5-13.1)	2019 (13.4)	15.5 (14.8-16.2)
Age 55-69 y								
Luminal A^b^	71 194 (76.7)	207.9 (206.4-209.5)	9260 (62.5)	164.3 (160.9-167.7)	8324 (72.7)	157.1 (153.7-160.5)	10 032 (72.3)	147.0 (144.1-149.9)
Luminal B^c^	9242 (10.0)	27.1 (26.5-27.6)	1720 (11.6)	30.3 (28.8-31.7)	1357 (11.9)	25.5 (24.2-26.9)	1626 (11.7)	23.4 (22.2-24.5)
ERBB2 enriched^d^	3841 (4.1)	11.3 (10.9-11.6)	849 (5.7)	14.9 (13.9-15.9)	809 (7.1)	15.2 (14.1-16.2)	775 (5.6)	11.1 (10.3-11.9)
Triple negative^e^	8562 (9.2)	25.1 (24.5-25.6)	2997 (20.2)	52.6 (50.8-54.6)	955 (8.3)	18 (16.9-19.2)	1443 (10.4)	20.9 (19.8-22)
Age 70-84 y								
Luminal A^b^	45 021 (80.9)	268.0 (265.6-270.5)	4748 (70.5)	214.1 (208.0-220.3)	3764 (78.5)	161.9 (156.7-167.1)	4629 (78.4)	173.6 (168.6-178.7)
Luminal B^c^	4258 (7.7)	25.3 (24.6-26.1)	564 (8.4)	25.4 (23.4-27.6)	394 (8.2)	17 (15.3-18.7)	506 (8.6)	19 (17.4-20.7)
ERBB2 enriched^d^	1663 (3.0)	9.9 (9.4-10.4)	325 (4.8)	14.7 (13.2-16.4)	200 (4.2)	8.6 (7.5-9.9)	232 (3.9)	8.7 (7.7-10.0)
Triple negative^e^	4692 (8.4)	27.8 (27.0-28.6)	1100 (16.3)	49.6 (46.7-52.6)	438 (9.1)	18.8 (17.1-20.6)	541 (9.2)	20.2 (18.5-22)

^a^ Per 100 000 women and age-adjusted to the 2000 US Standard Population (5-year age groups).

^b^ Indicates with hormone receptor expression and without ERBB2 expression.

^c^ Indicates with hormone receptor and ERBB2 expression.

^d^ Indicates without hormone receptor expression and with ERBB2 expression.

^e^ Indicates without hormone receptor or ERBB2 expression.

**Figure 1.**
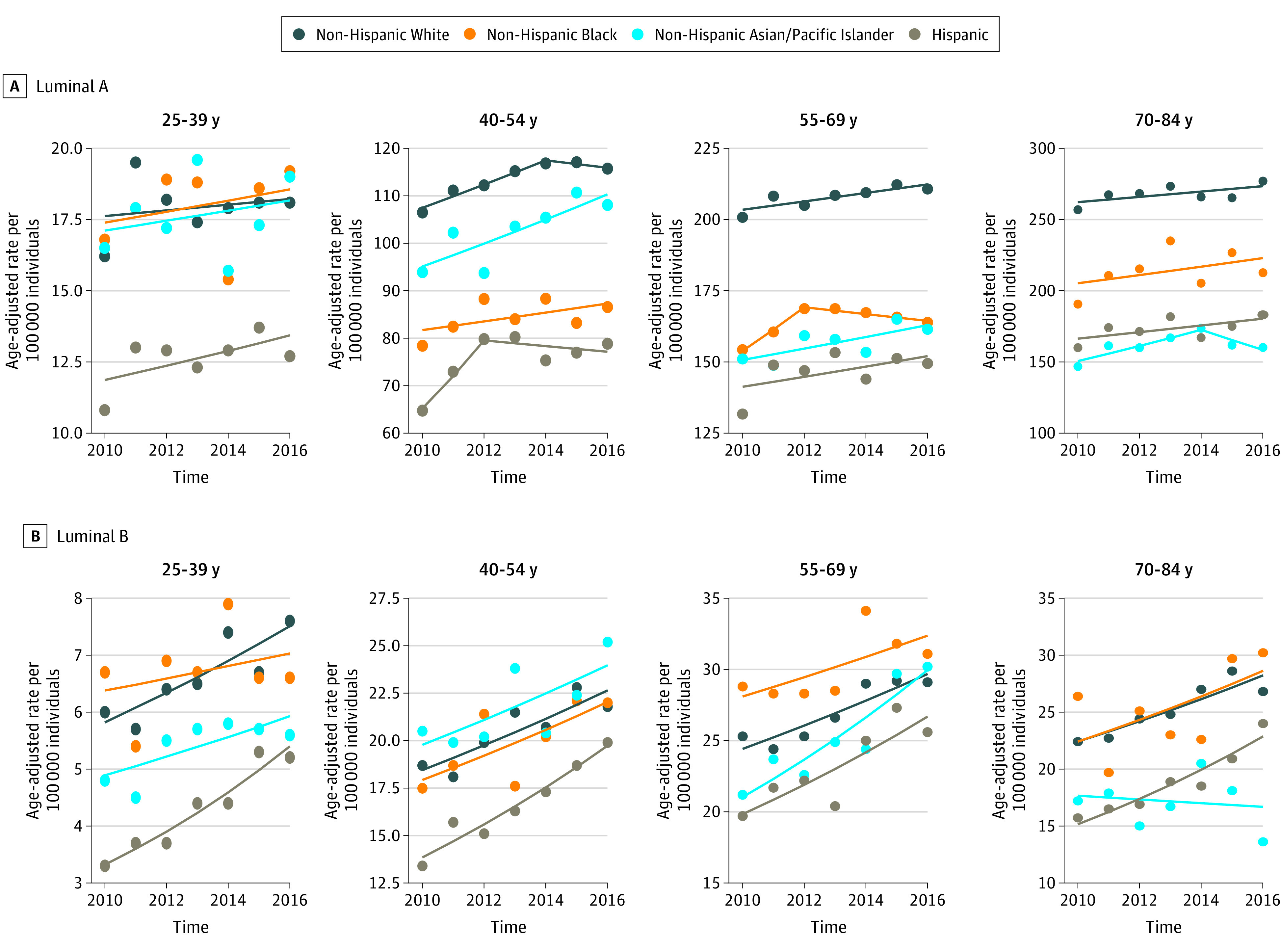
Incidence of Luminal A and B Breast Cancer Per 100 000 Women Stratified by Race/Ethnicity and Age Group Luminal A indicates hormone receptor–positive and ERBB2-negative; luminal B, hormone receptor–positive and ERBB2 positive.

**Figure 2.**
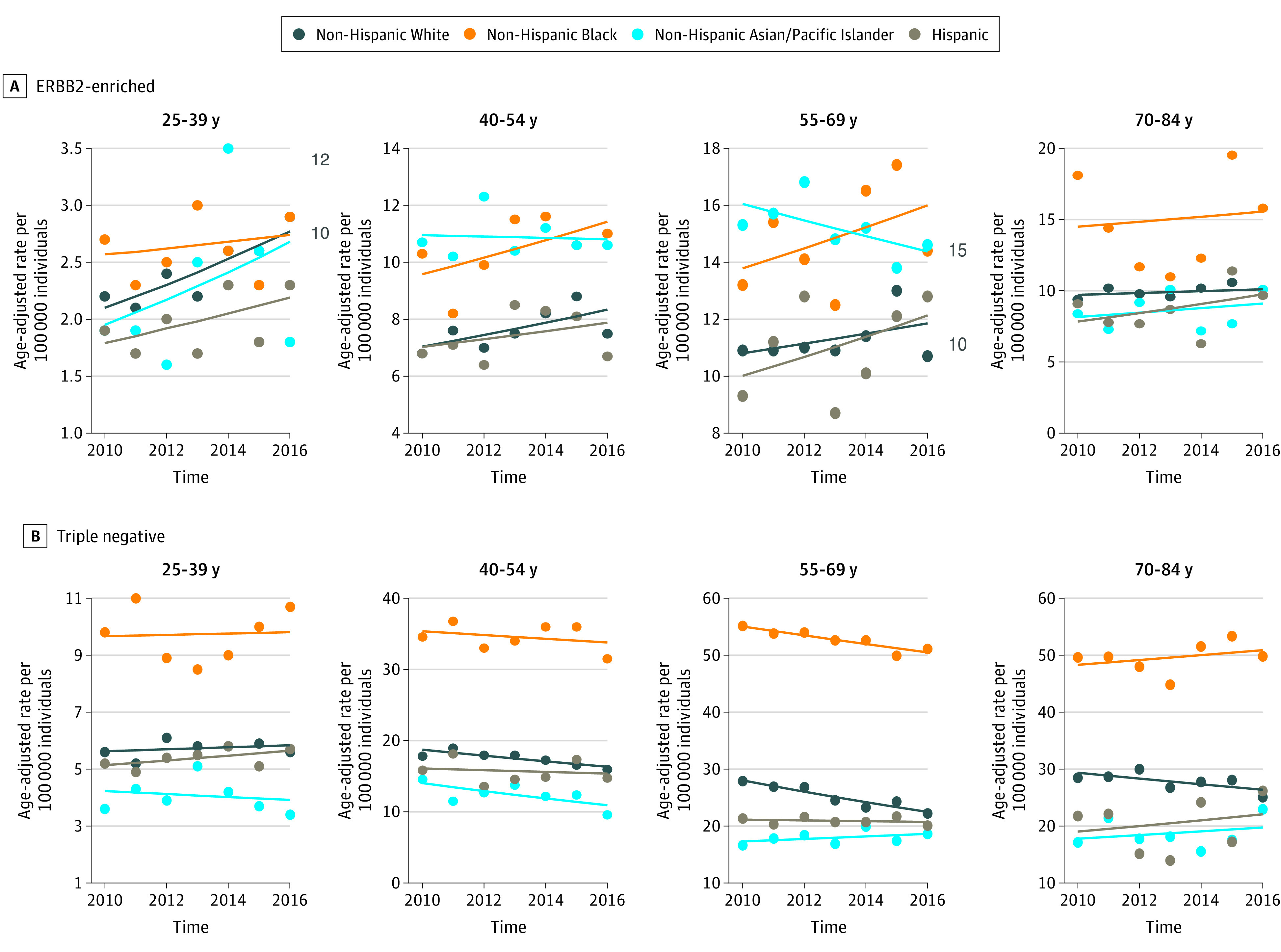
Incidence of ERBB2-Enriched and Triple-Negative Breast Cancer Per 100 000 Women Stratified by Race/Ethnicity and Age Group ERBB2-enriched indicates hormone receptor–negative and ERBB2-positive; triple-negative, hormone receptor–negative and ERBB2-negative.

Annual age-standardized breast cancer molecular subtype-specific incidence rates by age and racial/ethnic groups are displayed in Figure 1 and Figure 2, and the APCs in incidence rates along with 95% CIs are provided in Table 2. Luminal A breast cancer incidence rates increased for non-Hispanic White women aged 40 to 54 years old by 2.3% (95% CI, 0.3% to 4.2%) annually until 2014; the slope of the remaining trend line (ie, from 2014-2016) was not statistically significantly different from 0 in this group, indicating stable rates for that segment of time (Table 2; Figure 1A). Incidence rates for luminal A breast cancer also increased for non-Hispanic Black women aged 55 to 69 years by 4.9% (95% CI, 4.0% to 5.7%) annually until 2012, with a subsequent decline from 2013 to 2016 (APC, –0.7%; 95% CI, –0.9% to –0.5%) (Table 2, Figure 1A). There was also an increase in luminal A incidence rates for non-Hispanic Asian/Pacific Islander women aged 40 to 54 years from 2010 to 2016 (APC, 2.5%; 95% CI, 0.6% to 4.5%).

**Table 2.** Annual Percent Change Age-Standardized Breast Cancer Incidence Rates per 100 000 Women Stratified by Race/Ethnicity, Age Group, and Molecular Subtype From 2010 to 2016

Subtype	Annual percent change (95% CI)			
	Non-Hispanic White	Non-Hispanic Black	Non-Hispanic Asian/Pacific Islander	Hispanic
Age 25-39 y				
Luminal A^a^	0.6 (–2.4 to 3.6)	1.1 (–2.8 to 5.1)	1.0 (–2.9 to 5.1)	2.1 (–1.1 to 5.3)
Luminal B^b^	4.3 (1.5 to 7.2)	–1.6 (–3.8 to 7.5)	3.2 (–0.1 to 6.7)	8.4 (5.8 to 11.2)
ERBB2 enriched^c^	4.7 (1.5 to 8.0)	1.1 (–4.2 to 6.6)	5.4 (–8.6 to 21.6)	3.4 (–2.3 to 9.5)
Triple negative^d^	0.6 (–1.9 to 3.2)	0.2 (–4.8 to 5.5)	–1.3 (–8.6 to 6.6)	1.6 (–1.1 to 4.3)
Age 40-54 y				
Luminal A^a^	2.3 (0.3 to 4.2)^e^	1.1 (–0.8 to 3.0)	2.5 (0.6 to 4.5)	–0.8 (–6.8 to 5.7)^e^
Luminal A, includes data for 2010-2012^a^	–0.7 (–6.7 to 5.8)			10.5 (–11.4 to 37.7)
Luminal B^b^	3.5 (1.4 to 5.6)	3.5 (–0.2 to 7.3)	3.3 (–0.2 to 6.8)	6.1 (4.2 to 8.0)
ERBB2^c^ enriched	2.8 (–0.9 to 6.8)	3.0 (–2.2 to 8.4)	–0.2 (–3.6 to 3.3)	1.9 (–3.8 to 7.9)
Triple negative^d^	–2.3 (–3.8 to –0.7)	–0.8 (–3.5 to 2.0)	–4.1 (–9.2 to 1.2)	–0.8 (–6.1 to 4.9)
Age 55-69 y				
Luminal A^a^	0.7 (0.2 to 1.2)	–0.7 (–0.9 to –0.5^e^	1.3 (0.0 to 2.6)	1.2 (–0.8 to 3.3)
Luminal A, includes data for 2010-2012^a^		4.9 (4.0 to 5.7)		
Luminal B^b^	3.3 (1.6 to 5.0)	2.4 (–0.7 to 5.5)	6.1 (3.2 to 9.0)	5.1 (1.5 to 8.8)
ERBB2 enriched^c^	1.6 (–2.0 to 5.3)	2.5 (–3.4 to 8.7)	–1.8 (–4.3 to 0.8)	3.3 (–3.8 to 10.9)
Triple negative^d^	–3.6 (–5.1 to –2.1)	–1.4 (–2.2 to –0.7)	1.3 (–1.8 to 4.4)	–0.3 (–1.9 to 1.3)
Age 70-84 y				
Luminal A^a^	0.7 (–0.3 to 1.7)	1.4 (–1.8 to 4.6)	3.5 (–2.4 to 9.7)^e^	1.4 (–0.6 to 3.3)
Luminal A, includes data for 2010-2012^a^			–4.2 (–18.9 to 13.2)	
Luminal B^b^	3.9 (1.9 to 6.0)	4.2 (–2.2 to 10.9)	–1.0 (–8.0 to 6.6)	7.1 (4.6 to 9.6)
ERBB2 enriched^c^	0.7 (–1.5 to 2.8)	1.2 (–9.3 to 12.9)	1.8 (–5.5 to 9.8)	3.7 (–5.1 to 13.3)
Triple negative^d^	–1.8 (–3.9 to 0.5)	0.9 (–1.8 to 3.6)	1.8 (–5.2 to 9.2)	2.5 (–8.5 to 14.8)

^a^ Indicates with hormone receptor expression and without ERBB2 expression.

^b^ Indicates with hormone receptor and ERBB2 expression.

^c^ Indicates without hormone receptor expression and with ERBB2 expression.

^d^ Indicates without hormone receptor or ERBB2 expression.

^e^ Includes data from 2010 to 2014.

Luminal B, ERBB2-enriched, and triple-negative breast cancer incidence rates had no statistically significant changes in the slope of the trends (ie, inflection point in the trend) during the time period; however, there were significant changes in the rates over the entire time period from 2010 to 2016. Luminal B breast cancer incidence rates statistically significantly increased in all age groups for non-Hispanic White women (age 25-39 years: APC, 4.3%; 95% CI, 1.5% to 7.2%; age 40-54 years: APC, 3.5%; 95% CI, 1.4% to 5.6%; age 55-69 years: APC, 3.3%; 95% CI, 1.6% to 5.0%; age 70-84 years: APC, 3.9%; 95% CI, 1.9% to 6.0%) and Hispanic women (age 25-39 years: APC, 8.4%; 95% CI, 5.8% to 11.2%; age 40-54 years: APC, 6.1%; 95% CI, 4.2% to 8.0%; age 55-69 years: APC, 5.1%; 95% CI, 1.5% to 8.8%; age 70-84 years: APC, 7.1%; 95% CI, 4.6% to 9.6%) (Table 2 and Figure 1B), with the largest increases for each of these racial/ethnic groups observed for women aged 25 to 39-years. There was also a 6.1% (95% CI, 3.2% to 9.0%) annual increase in luminal B breast cancer incidence rates for non-Hispanic Asian/Pacific Islander women aged 55 to 69 years (Table 2 and Figure 1B).

Incidence rates for ERBB2-enriched breast cancer increased by 4.7% (95% CI, 1.5% to 8.0%) annually for non-Hispanic White women aged 25 to 39 years (Table 2 and Figure 2A). There were no statistically significant changes in ERBB2-enriched incidence rates for any other racial/ethnic and age group combinations, but the CIs for some of the estimates were wide. Triple-negative breast cancer incidence rates decreased by 2.3% (95% CI, –3.8% to –0.7%) annually in non-Hispanic White women aged 40 to 54 years, by 3.6% (95% CI, –5.1% to –2.1%) annually in non-Hispanic White women aged 55 to 69 years, and by 1.4% (95% CI, –2.2% to –0.7%) annually in non-Hispanic Black women (Table 2 and Figure 2B).

## Discussion

This cross-sectional study found that between 2010 and 2016, hormone receptor–positive breast cancer subtypes (ie, luminal A and B) increased for many racial/ethnic and age groups, with the largest and most consistent increases observed for luminal B breast cancer in non-Hispanic White and Hispanic women. Luminal A breast cancer incidence rates increased for non-Hispanic White and non-Hispanic Asian/Pacific Islander women aged 40 to 69 years and for non-Hispanic Black women aged 55 to 69 years, although the incidence rates began to decline for non-Hispanic Black women by 2012. Trends for hormone receptor–negative breast cancer subtypes (ie, ERBB2-enriched and triple negative) were mixed; ERBB2-enriched breast cancer increased for non-Hispanic White women aged 25 to 30 years, while triple-negative breast cancer decreased for non-Hispanic White women aged 40 to 69 years, and non-Hispanic Black women aged 55 to 69 years.

Our results are consistent with another population-based study^2^ that found an increase in hormone receptor–positive breast cancer and a decline in hormone receptor–negative breast cancer, which occurred to varying degrees for all racial/ethnic groups among women younger than 50 years and those 50 years or older. By further considering ERBB2 status and smaller age groups, we found differing trends in hormone receptor–positive and hormone receptor–negative breast cancers based on positive ERBB2 expression, enabling us to identify additional racial/ethnic and age group variation in trends. Specifically, we found that the increases for hormone receptor–positive tumors were driven by increases in luminal A subtype in midlife non-Hispanic White and non-Hispanic Asian/Pacific Islander women and increases in luminal B subtype that spanned across all age groups for non-Hispanic White and Hispanic women. Similarly, considering the ERBB2-enriched subtype separately from the triple-negative subtype, we were able to confirm that declines in hormone receptor–negative breast cancers were restricted to the triple-negative subtype in midlife non-Hispanic White and non-Hispanic Black women and did not include the ERBB2-enriched subtype, for which an increase in young non-Hispanic White women was observed.

Reasons for age and racial/ethnic group variations in subtype-specific breast cancer incidence are not adequately known. Increasing evidence, including a 2018 pooled analysis of 9 prospective cohorts,^9^ suggests variations in the direction and magnitude of associations of known breast cancer risk factors (eg, parity and age at first birth, menarche, and menopause) with breast cancer subtypes, indicating heterogeneity in breast cancer origins; variations in the prevalence of breast cancer risk factors across race/ethnicity and birth cohorts have been documented.^2,9,19,20,21^ Thus, differences in the distribution of population-level breast cancer risk factors by race/ethnicity and birth cohorts may drive subtype-specific breast cancer incidence rates, and may partially reflect racial/ethnic and age incidence trends in breast cancer at the molecular level,^2,21,22^ although this requires further empirical confirmation. Moreover, changes in incidence trends for particular subtypes, such as ERBB2-positive breast cancers, that are consistent across all age groups or that are present for younger age groups who are not likely participating in breast cancer screening suggest a real change in risk for those subtypes rather than greater detection through increasing screening over time.

Strengths of this study include the large study population with a wide range of age groups. Additionally, this cross-sectional study included detailed and consistent assessment and reporting of data for characterizing molecular subtypes of breast cancer for the time period covered in this analysis.

### Limitations

There are some limitations of this study. As data for ERBB2 status were not collected by cancer registries until 2010, we restricted this analysis to 2010 to 2016, the most recent years for which more complete data were available. The relatively short period of time did not allow for detection of longer-term changes in the incidence rates and allowed for identifying only 1 possible inflection point in the incidence trend in the Joinpoint analysis. The small sample of American Indian and Alaska Native women did not allow for including this population in Joinpoint analysis, and the small sample of women diagnosed with ERBB2-enriched breast cancer subtype resulted in imprecise estimates. Replication of our analysis with additional years as well as a larger sample of American Indian and Alaska Native women is warranted. Our analysis excluded approximately 7% of patients identified with invasive breast cancer in 2010 to 2016 owing to missing tumor marker status data. The distribution of missing tumor status data was similar across age and race/ethnicity groups, although a slightly larger number of Hispanic women overall and women aged 70 to 84 years in each race/ethnic group had unknown tumor marker data.^5^ As a result, we cannot rule out that the observed rates may be overestimated or underestimated for different groups.

## Conclusions

The findings of this cross-sectional study suggest that overall breast cancer trends mask differences that may exist across molecular subtypes by age and racial/ethnic groups. In recent years, luminal A and luminal B breast cancer incidence has increased, whereas triple-negative breast cancer trends have gradually declined for certain age and racial/ethnic groups. Our results highlight the heterogeneity of these trends and underscore the importance of surveillance of breast cancer trends by clinically relevant subgroups to guide breast cancer prevention and control efforts.

## Notes

Supplement. **eTable.** Number and Incidence Per 100 000 Women of Breast Cancer Stratified by Race/Ethnicity, Age Group, and Molecular Subtype
