# Supplementary material for: Incidence Trends of Breast Cancer Molecular Subtypes by Age and Race/Ethnicity in the US From 2010 to 2016
Source: JAMA Netw Open. 2020 Aug 17;3(8):e2013226. doi: 10.1001/jamanetworkopen.2020.13226 (PMC7431997; doi:10.1001/jamanetworkopen.2020.13226)
Supplement: Supplement. — eTable. Number and Incidence Per 100 000 Women of Breast Cancer Stratified by Race/Ethnicity, Age Group, and Molecular Subtype [file jamanetwopen-3-e2013226-s001.pdf]

Supplementary Online Content

Acheampong T, Kehm RD, Terry MB, Argov EL, Tehranifar P. Incidence trends of breast cancer molecular subtypes by age and race/ethnicity in the US from 2010 to 2016. *JAMA Netw Open*. 2020;3(8):e2013226. doi:10.1001/jamanetworkopen.2020.13226

eTable. Number and Incidence Per 100 000 Women of Breast Cancer Stratified by Race/Ethnicity, Age Group, and Molecular Subtype

This supplementary material has been provided by the authors to give readers additional information about their work.

© 2020 Acheampong T et al. *JAMA Network Open.*

eTable. Number and Incidence Per 100 000 Women of Breast Cancer Stratified by Race/Ethnicity, Age Group, and Molecular Subtype

	Incidence Rate for 2010-2016 per 100,000 (95% CI)	N (%)
Non-Hispanic white		
25-39-year olds		
HR+/HER2+ (Luminal B)	6.6 (6.3, 6.9)	1,845 (19.2%)
HR-/HER2+ (HER2 enriched)	2.4 (2.2, 2.6	676 (7.0%)
HR+/HER2- (Luminal A)	17.9 (17.4, 18.4)	4,950 (51.4%)
HR-/HER2- (Triple Negative)	5.7 (5.4, 6.0)	1611 (16.7%)
Unknown	2.0 (1.8, 2.1)	545 (5.7%)
40-54-year olds		
HR+/HER2+ (Luminal B)	20.4 (20.0, 20.9)	7,430 (11.9%)
HR-/HER2+ (HER2 enriched)	7.6 (7.3, 7.9)	2,826 (4.5%)
HR+/HER2- (Luminal A)	113.3 (112.2, 114.5)	41,930 (67.4%)
HR-/HER2- (Triple Negative)	17.5 (17.1, 17.9)	6,351 (10.2%)
Unknown	10.0 (9.7, 10.4)	3,704 (6.0%)
55-69-year olds		
HR+/HER2+ (Luminal B)	27.1 (26.5, 27.6)	9,242 (9.3%)
HR-/HER2+ (HER2 enriched)	11.3 (10.9, 11.6)	3,841 (3.9%)
HR+/HER2- (Luminal A)	207.9 (206.4, 209.5)	71,194 (71.7%)
HR-/HER2- (Triple Negative)	25.1 (24.5, 25.6)	8,562 (8.6%)
Unknown	19.0 (18.6, 19.5)	6,511 (6.6%)
70-84-year olds		
HR+/HER2+ (Luminal B)	25.3 (24.6, 26.1)	4258 (7.1%)
HR-/HER2+ (HER2 enriched)	9.9 (9.4, 10.4)	1663 (2.8%)
HR+/HER2- (Luminal A)	268 (265.6, 270.5)	45021 (75.0%)
HR-/HER2- (Triple Negative)	27.8 (27.0, 28.6)	4692 (7.8%)
Unknown	25.9 (25.1, 26.7)	4379 (7.3%)
Non-Hispanic black		
25-39-year olds		
HR+/HER2+ (Luminal B)	6.7 (6.1, 7.3)	491 (17.0%)
HR-/HER2+ (HER2 enriched)	2.6 (2.3, 3.0)	192 (6.6%)
HR+/HER2- (Luminal A)	17.9 (17.0, 18.9)	1300 (44.9%)
HR-/HER2- (Triple Negative)	9.7 (9.0, 10.5)	711 (24.6%)
Unknown	2.7 (2.4, 3.1)	200 (6.9%)
40-54-year olds		
HR+/HER2+ (Luminal B)	19.9 (19.0, 20.9)	1597 (12.3%)
HR-/HER2+ (HER2 enriched)	10.4 (9.7, 11.2)	842 (6.5%)
HR+/HER2- (Luminal A)	84.4 (82.4, 86.5)	6783 (52.3%)
HR-/HER2- (Triple Negative)	34.6 (33.3, 35.9)	2788 (21.5%)
Unknown	12.0 (11.2, 12.7)	966 (7.4%)
55-69-year olds		
HR+/HER2+ (Luminal B)	30.3 (28.8, 31.7)	1720 (10.7%)
HR-/HER2+ (HER2 enriched)	14.9 (13.9, 15.9)	849 (5.3%)
HR+/HER2- (Luminal A)	164.3 (160.9, 167.7)	9260 (57.7%)
HR-/HER2- (Triple Negative)	52.6 (50.8, 54.6)	2997 (18.7%)

© 2020 Acheampong T et al. *JAMA Network Open.*

Unknown	21.4 (20.2, 22.7)	1210 (7.5%)
70-84-year olds		
HR+/HER2+ (Luminal B)	25.4 (23.2, 27.6)	564 (7.7%)
HR-/HER2+ (HER2 enriched)	14.7 (13.2, 16.4)	325 (4.4%)
HR+/HER2- (Luminal A)	214.1 (208.0, 220.3)	4748 (64.5%)
HR-/HER2- (Triple Negative)	49.6 (46.7, 52.6)	1100 (14.9%)
Unknown	28.2 (26.0, 30.5)	623 (8.5%)
Non-Hispanic API		
25-39-year olds		
HR+/HER2+ (Luminal B)	5.4 (4.9, 6.0)	405 (17.3%)
HR-/HER2+ (HER2 enriched)	2.3 (1.9, 2.6)	169 (7.2%)
HR+/HER2- (Luminal A)	17.6 (16.7, 18.6)	1307 (55.9%)
HR-/HER2- (Triple Negative)	4.0 (3.6, 4.5)	301 (12.9%)
Unknown	2.1 (1.8, 2.4)	157 (6.7%)
40-54-year olds		
HR+/HER2+ (Luminal B)	21.8 (20.8, 22.9)	1611 (13.7%)
HR-/HER2+ (HER2 enriched)	10.9 (10.1, 11.7)	809 (6.9%)
HR+/HER2- (Luminal A)	102.7 (100.4, 105.0)	7564 (64.5%)
HR-/HER2- (Triple Negative)	12.3 (11.5, 13.1)	902 (7.7%)
Unknown	11.4 (10.6, 12.2)	843 (7.2%)
55-69-year olds		
HR+/HER2+ (Luminal B)	25.5 (24.2, 26.9)	1357 (11.0%)
HR-/HER2+ (HER2 enriched)	15.2 (14.1, 16.2)	809 (6.5%)
HR+/HER2- (Luminal A)	157.1 (153.7, 160.5)	8324 (67.4%)
HR-/HER2- (Triple Negative)	18.0 (16.9, 19.2)	955 (7.7%)
Unknown	17.1 (16.0, 18.3)	907 (7.3%)
70-84-year olds		
HR+/HER2+ (Luminal B)	17.0 (15.3, 18.7)	394 (7.5%)
HR-/HER2+ (HER2 enriched)	8.6 (7.5, 9.9)	200 (3.8%)
HR+/HER2- (Luminal A)	161.9 (156.7, 167.1)	3764 (72.1%)
HR-/HER2- (Triple Negative)	18.8 (17.1, 20.6)	438 (8.4%)
Unknown	18.2 (16.5, 20.0)	423 (8.1%)
Hispanic		
25-39-year olds		
HR+/HER2+ (Luminal B)	4.3 (4.0, 4.6)	648 (16.4%)
HR-/HER2+ (HER2 enriched)	2 (1.8, 2.2)	299 (7.6%)
HR+/HER2- (Luminal A)	12.6 (12.1, 13.2)	1897 (48.1%)
HR-/HER2- (Triple Negative)	5.4 (5.0, 5.7)	814 (20.6%)
Unknown	1.9 (1.7, 2.1)	286 (7.3%)
40-54-year olds		
HR+/HER2+ (Luminal B)	16.7 (16.0, 17.4)	2175 (13.3%)
HR-/HER2+ (HER2 enriched)	7.4 (7.0, 7.9)	965 (5.9%)
HR+/HER2- (Luminal A)	75.7 (74.2, 77.2)	9855 (60.2%)
HR-/HER2- (Triple Negative)	15.5 (14.8, 16.2)	2019 (12.3%)
Unknown	10.5 (9.9, 11.1)	1367 (8.3%)
55-69-year olds		
HR+/HER2+ (Luminal B)	23.4 (22.2, 24.5)	1626 (10.7%)
HR-/HER2+ (HER2 enriched)	11.1 (10.3, 11.9)	775 (5.1%)
HR+/HER2- (Luminal A)	147.0 (144.1, 149.9)	10032 (65.8%)
HR-/HER2- (Triple Negative)	20.9 (19.8, 22.0)	1443 (9.5%)

© 2020 Acheampong T et al. *JAMA Network Open.*

Unknown	20.0 (18.9, 21.1)	1365 (9.0%)
70-84-year olds		
HR+/HER2+ (Luminal B)	19.0 (17.4, 20.7)	506 (7.7%)
HR-/HER2+ (HER2 enriched)	8.7 (7.7, 10.0)	232 (3.5%)
HR+/HER2- (Luminal A)	173.6 (168.6, 178.7)	4629 (70.5%)
HR-/HER2- (Triple Negative)	20.2 (18.5, 22.0)	541 (8.2%)
Unknown	24.9 (23.0, 26.9)	661 (10.1%)

© 2020 Acheampong T et al. *JAMA Network Open.*
